# Soy isoflavones increase preprandial peptide YY (PYY), but have no effect on ghrelin and body weight in healthy postmenopausal women

**DOI:** 10.1186/1477-5751-5-11

**Published:** 2006-08-14

**Authors:** Martin O Weickert, Manja Reimann, Bärbel Otto, Wendy L Hall, Katherina Vafeiadou, Jesper Hallund, Marika Ferrari, Duncan Talbot, Francesco Branca, Susanne Bügel, Christine M Williams, Hans-Joachim Zunft, Corinna Koebnick

**Affiliations:** 1 German Institute of Human Nutrition Potsdam-Rehbruecke, Nuthetal, Germany; 2 Dept. of Endocrinology, Diabetes and Nutrition, Charité-University-Medicine, Berlin, Germany; 3 School for Physiology, Nutrition and Consumer Sciences, North-West University Potchefstroom-Campus, Potchefstroom, South Africa; 4 Medical Dept., University Hospital Innenstadt, Munich, Germany; 5 School of Food Biosciences, University of Reading, Reading, UK; 6 The Royal Veterinary & Agricultural University, Research Dept. of Human Nutrition, Centre for Advanced Food Studies, Frederiksberg, Denmark; 7 Istituto Nazionale di Ricerca per gli Alimenti e la Nutrizione, Rome, Italy; 8 Unilever Corporate Research, Colworth House, Sharnbrook, Bedfordshire, UK; 9 Dept. of Preventive Medicine, University of Southern California, Los Angeles, USA

## Abstract

**Background:**

Soy isoflavones show structural and functional similarities to estradiol. Available data indicate that estradiol and estradiol-like components may interact with gut "satiety hormones" such as peptide YY (PYY) and ghrelin, and thus influence body weight. In a randomized, double-blind, placebo-controlled, cross-over trial with 34 healthy postmenopausal women (59 ± 6 years, BMI: 24.7 ± 2.8 kg/m^2^), isoflavone-enriched cereal bars (50 mg isoflavones/day; genistein to daidzein ratio 2:1) or non-isoflavone-enriched control bars were consumed for 8 weeks (wash-out period: 8-weeks). Seventeen of the subjects were classified as equol producers. Plasma concentrations of ghrelin and PYY, as well as energy intake and body weight were measured at baseline and after four and eight weeks of each intervention arm.

**Results:**

Body weight increased in both treatment periods (isoflavone: 0.40 ± 0.94 kg, P < 0.001; placebo: 0.66 ± 0.87 kg, P = 0.018), with no significant difference between treatments. No significant differences in energy intake were observed (P = 0.634). PYY significantly increased during isoflavone treatment (51 ± 2 pmol/L vs. 55 ± 2 pmol/L), but not during placebo (52 ± 3 pmol/L vs. 50 ± 2 pmol/L), (P = 0.010 for treatment differences, independent of equol production). Baseline plasma ghrelin was significantly lower in equol producers (110 ± 16 pmol/L) than in equol non-producers (162 ± 17 pmol/L; P = 0.025).

**Conclusion:**

Soy isoflavone supplementation for eight weeks did not significantly reduce energy intake or body weight, even though plasma PYY increased during isoflavone treatment. Ghrelin remained unaffected by isoflavone treatment. A larger and more rigorous appetite experiment might detect smaller differences in energy intake after isoflavone consumption. However, the results of the present study do not indicate that increased PYY has a major role in the regulation of body weight, at least in healthy postmenopausal women.

## Background

Several intervention studies in humans and animals suggest that consumption of soy and isoflavone-rich soy protein may decrease body weight [1,2]. Postmenopausal women with relatively high isoflavone consumption in their normal diet showed an inverse association with obesity in a cross-sectional study [3]. However, the protein content of soy may be at least partly responsible for the observed effects, and the contribution of soy isoflavones such as genistein and daidzein remains uncertain [4,5]. There is some evidence that may link isolated isoflavone consumption *per se *to the regulation of body weight. Soy isoflavones show functional and structural similarities to estradiol [6], mainly by binding to the estrogen receptor β [7]. In ovariectomized mice, loss of circulating estrogen increases body weight and fat mass, and this is reversed by estrogen replacement [8,9]. Similar effects have been observed in ovariectomized mice treated with oral genistein [10,11]. Even though the role of postmenopausal hormone replacement in modulating body weight is controversial [12,13], some studies indicate that the activation of the estrogen receptor interferes with the regulation of gut hormones commonly thought to be involved in the regulation of food intake. Food intake decreases during the high estrogen period in the estrous cycle in rats, and estradiol replacement in ovariectomized rats increased the satiating effect of the gut hormone cholecystokinin [14]. Estrogen replacement in hysterectomized postmenopausal women also increased peripheral concentrations of the orexigenic gut hormone ghrelin [15]. Peptide YY (PYY), a member of the neuropeptide Y (NPY) family and another gut derived "satiety hormone", is assumed to have potent anorexigenic properties, with potential therapeutic use in obese humans [16]. Human PYY has been shown to be regulated in a gender specific manner, with higher PYY secretion in females than in males [17]. Treatment of ovariectomized rodents with estradiol increases the number of receptors of several neuropeptides, including NPY receptors in the brain [18]. To date, potential effects of isolated isoflavones on PYY have not been reported, and only one study investigated effects of isolated isoflavones on total ghrelin concentrations [19]. In addition, no long-term randomized controlled studies have investigated whether changes in PYY concentrations influence food intake in free living humans. The hypothesis of the present study was that estradiol-like properties of isolated isoflavones may influence PYY and ghrelin, and thus energy intake and body weight.

## Results

Results of biomarkers are given as postabsorptive concentrations, measured after a standardized low-fat evening meal and after 12 h overnight fasts. P values for PYY, glucose, insulin and ghrelin are given for the treatment effect within a linear mixed model. Differences from baseline were used as response variable after adjustment for changes in BMI.

### Dietary intake and body weight

Dietary intake was assessed at baseline and after four weeks of each intervention arm. Macronutrient intake at baseline was 15% of energy as protein, 34% as fat, and 47% as carbohydrate. Even though participants were instructed to replace snacks by the cereal bars, body weight increased moderately, but significantly, during both intervention periods (placebo + 0.66 ± 0.87 kg, (P = 0.018); isoflavones + 0.40 ± 0.94 kg, (P < 0.001)). There was no significant difference in body weight and body mass index (P > 0.331) between treatments (table 1). There were no significant differences in energy intake or macronutrient intake, both across the treatments and compared to baseline (wk4 - wk0; interaction treatment vs. time, P = 0.634).

**Table 1:** Plasma PYY, body weight, and urinary isoflavone concentrations in postmenopausal women, at baseline (t0) and after 8 weeks (t8) of isoflavone or placebo consumption (n = 34)

	**Isoflavones**		**Placebo**		**P value^1^**
	t0	t8	t0	t8	
Total PYY (pmol/L)	51 ± 2	55 ± 2	52 ± 3	50 ± 2	0.010
BMI (kg/m^2^)	24.5 ± 2.7	24.6 ± 2.7	24.5 ± 2.8	24.7 ± 2.8	0.331
Glucose (mmol/L)	5.7 ± 0.1	5.6 ± 0.1	5.7 ± 0.1	5.6 ± 0.1	0.641
Insulin (pmol/L)	37 ± 3	39 ± 3	38 ± 3	34 ± 3	0.231
Total ghrelin (pmol/L)	129 ± 12	131 ± 12	133 ± 13	123 ± 11	0.297

Data are given as mean ± SEM except for BMI which is presented as mean ± SD. BMI = body mass index.

^1 ^P values are shown for the treatment effect within a linear mixed model. Differences from baseline were used as response variable after adjustment for changes in BMI.

### Effect of isoflavone consumption on PYY

During isoflavone consumption, PYY concentrations increased by eight percent, and during placebo consumption, PYY concentrations decreased by four percent (P = 0.010 for treatment differences) (table 1).

Changes in PYY levels were independent of changes in BMI, and were negatively correlated with baseline PYY (r = -0.67; P < 0.001). PYY concentrations were not significantly different between equol producers and equol non-producers.

### Effect of isoflavone consumption on ghrelin

At baseline, ghrelin was significantly lower in equol producers (110 ± 16 pmol/L) than in equol non-producers (162 ± 17 pmol/L); (P = 0.025), independent of BMI. However, isoflavone treatment did not affect ghrelin concentrations (table 1).

### Other parameters

During isoflavone treatment, urinary genistein and daidzein excretion increased 15 fold and 24 fold, respectively. There was no significant increase in urinary genistein and daidzein concentrations following placebo treatment. According to the cut-offs used in the present study [20], 50 percent of the participants (n = 17) were classified as equol producers. Equol production increased 35 fold in equol producers during the active treatment, compared to an 1.7 fold increase in equol non-producers (table 2).

**Table 2:** Urinary isoflavone concentrations in postmenopausal women at baseline (t0) and week 8 (t8) of isoflavone and placebo arms (n = 34)

	**Isoflavones**		**Placebo**		***P *value^1^**
	t0	t8	t0	t8	
Genistein (nmol/L)	808 ± 134	12266 ± 852	655 ± 68	647 ± 73	<0.001
Daidzein (nmol/L)	334 ± 88	8146 ± 651	315 ± 82	223 ± 40	<0.001
Equol, (nmol/L)	154 ± 12	3040 ± 713	162 ± 15	198 ± 23	<0.001
Equol producers (n = 17)	164 ± 11	5834 ± 749	179 ± 16	237 ± 29	<0.001
Equol non-producers (n = 17)	143 ± 14	245 ± 16	145 ± 13	160 ± 12	<0.001

Data are given as mean ± SEM.

^1 ^P values are shown for the treatment effect within a linear mixed model. Differences from baseline were used as response variable.

Plasma glucose and insulin were unaffected by isoflavone treatment (table 1).

### Power analysis

A between-treatment difference in body weight > 500 g after 8 weeks intervention was assumed to be relevant. The estimated power of this study was 94 percent to detect a difference of 500 ± 900 g in body weight between treatments with a sample size of 34 subjects and a significance level of 0.05.

## Discussion

Consumption of soy derived food rich in isoflavones has been suggested to have favorable effects on energy intake and body weight [5]. However, soy derived food is also rich in protein, and an increased protein intake might be responsible for the observed effects. The potential contribution of isolated isoflavones to the regulation of energy and body weight remains uncertain. In the present study, isoflavone treatment for eight weeks did not significantly influence energy intake, macronutrient intake, or body weight, both across the treatments or compared to baseline. Isoflavone treatment did not affect preprandial ghrelin, which seems to be in contrast to results of a previous study that investigated effects of isoflavone treatment on ghrelin concentrations [19]. However, the observed differences in the mentioned study are mainly due to increased ghrelin concentrations in the placebo group rather than altered ghrelin concentrations after isoflavone intake. In addition, high within and between subject variations in preprandial ghrelin concentrations have been reported, which may lead to the detection of random effects rather than of true treatment effects [21]. Despite unchanged ghrelin concentrations during isoflavone treatment in the present study, the ability of producing equol, a gut bacterial metabolite of daidzein with higher binding affinity to estrogen receptors compared with its precursor [22], was associated with lower ghrelin concentrations at baseline. The capability to produce equol is greatly varying between individuals, with about 30–40% equol producers in the Western population [22]. It is speculated that equol producers may have an increased benefit from soy consumption [20,23]. However, even though the ability of producing equol was associated with lower ghrelin concentrations at baseline, ghrelin responses remained unaffected during isoflavone treatment in the present study. This indicates that equol may have a long-term suppressive effect on ghrelin concentrations, which probably will not respond to further and relatively short-term increases of equol concentrations. In contrast to unchanged ghrelin in the present study, isolated soy isoflavones significantly increased plasma PYY concentrations. Given the assumed potent anorexigenic properties of PYY [16], our data do not suggest a major role of PYY on the regulation of body weight. Notably, power analysis indicated that even a moderate difference in body weight between treatments was highly likely to be detected. Only moderate effects of PYY on the regulation of body weight may contribute to the explanation of controversial findings in the literature. In humans, short-term intravenous administration of PYY [3-36] in supraphysiological [24,25], but not in physiological doses [25] reduces appetite and food intake. Long-term studies in humans are not available to date. A two weeks continous PYY infusion in colectomized rats did not affect food intake and body weight [26], and data obtained from other animal studies are controversially discussed [27]. The duration of the present study may have been too short to detect relevant differences in body weight. However, most studies linking physiological ghrelin and PYY responses to food intake investigated the effects of only one meal. A relatively small but well performed study over 16 weeks did not show an effect of macronutrient intake and energy intake on preprandial ghrelin [21]. It needs, however, to be emphasized that food diaries, and not weighed food intake were used to assess energy intake in the present study, and a more rigorous appetite experiment might detect smaller effects. In addition, adaptation processes and counter regulatory responses in other satiety hormones than total ghrelin, such as acylated ghrelin, glucagon-like peptide (GLP-1), or cholecystokinin may have masked detectable differences in energy intake or body weight.

## Conclusion

Isoflavone treatment had no effect on energy intake and body weight, despite significantly increased preprandial PYY concentrations. The findings indicate that PYY is not a major factor in the regulation of body weight. Preprandial ghrelin was not affected by isoflavone consumption. The isoflavone contents are not likely to explain the observed beneficial effects of soy consumption on energy intake and body weight.

## Methods

### Subjects

This study was part of a multi-center intervention located in Frederiksberg (Denmark), Reading (UK), Rome (Italy), and Potsdam (Germany). Potential effects of isoflavones on gut satiety hormones and body weight were investigated in the German population. Thirty-six healthy postmenopausal women (age 59 ± 6 y, BMI 24.7 ± 2.8 kg/m^2^), defined as at least 12 months since the last menstrual cycle, were recruited by advertisement in the local media. Thirty-four of the subjects completed the intervention. One of the subjects was excluded because of a prolonged respiratory infection, the other one because of start of a treatment with an angiotensin-converting-enzyme inhibitor. None of the volunteers had used hormone replacement therapy for six months, antibiotics for three months, or isoflavone, vitamin, or mineral containing supplements for two months. All volunteers were non-smokers. Parameters of renal and liver function were within normal range. Subjects were classified as equol producers, when equol in a 24 h urine sample exceeded 936 nmol/liter during isoflavone treatment, which corresponds to an urinary equol excretion of > 0.45 mg/day [20]. The study protocol was approved by the Ethics Committee of the University of Potsdam, Germany. All volunteers gave written informed consent prior to the study.

### Study design

This was a randomized, double-blind, placebo-controlled, 2 × 8-wk crossover study, separated by an 8-wk washout period. Subjects were invited to the metabolic unit on 6 occasions (t0, t4, and t8 on each intervention arm), after 12-h overnight fasts. To exclude potential second-meal effects, a set low-fat evening meal (< 10 g fat) was consumed the evening before each of the study days. Recipes for the preparation of the meals were provided to the participants. Energy contents of the meals were comparable. Subjects were asked to consume two fruit cereal bars/d (Health & Diet Food, Manchester, UK), one in the morning and one in the afternoon, in addition to their normal diet. During the treatment period, cereal bars were enriched with 2 × 25 mg isoflavones/d, with a genistein to daidzein ratio of 2:1 ("Solgen 40", Solbar Plant Extracts, Ashdod, Israel). Thus, isoflavone intake in the treatment group of the present study was in the upper range of the daily isoflavone intake in traditional Asian diets (15 – 50 mg/d) [28]. The product was tested before packaging and during the study by HPLC, to ensure stability of the isoflavones [29]. Placebo did not contain any isoflavones. Each cereal bar (40 g) had an average nutrient content of energy (652 kJ); protein 2.6 g; carbohydrate 17.3 g; fat 8.5 g; fiber 1.8 g; sodium 0.012 g. Subjects perceived the isoflavone-enriched and placebo cereal bars as identical in taste and visual appearance. Habitual diet was assessed by estimated 3-d food records three times during the study. Diet diaries were completed at baseline (t0) and after 4 weeks (t4) of each intervention arm. All food records included two week days and one weekend day. Nutrient intake was calculated based on the German Food and Nutrient Data Base Bundeslebensmittelschlüssel BLS II.3 [30]. To avoid weight gain, subjects were advised to replace snacks with the cereal bars. Subjects kept daily records of cereal bar consumption and well-being in a study diary. Dietary compliance was further assessed by measurement of phytoestrogen concentrations in 24-hour urine [31], which was collected at start and end of each intervention period. Body weight was measured at each visit.

### Biochemical parameters

Blood was collected in ice-chilled EDTA tubes for the analysis of glucose, ghrelin, and PYY. Following centrifugation at 1600 g for 10 minutes at 4°C, aliquots were immediately frozen at -20°C until assayed. All samples from individual subjects were measured in the same assay. Immunoreactive total ghrelin was measured by a commercially available radioimmunoassay (Phoenix Pharmaceuticals, Mountain View, CA, USA), as previously described [32]. Immunoreactive total human PYY was measured by a commercially available radioimmunoassay (LINCO Research, Missouri, USA), using^125^I-labeled bioactive PYY as tracer and a PYY antiserum to determine the level of active PYY by the double antibody/PEG technique. The PYY antibody is raised in guinea pigs and recognizes both the PYY 1–36 and PYY 3–36 forms of human PYY. Intra- and inter-assay coefficient of variation was 5.3% and 7.0%, respectively. Insulin, and glucose, and urinary phytoestrogens (genistein, daidzein, equol) were analyzed as previously described [33].

### Statistical analyses

Data are given as mean ± SEM, anthropometric data are given as mean ± SD. Changes from baseline, e.g. week-8 compared to week-0 (t8-t0), were used as the dependent variables. Data were calculated as changes from baseline on the original scale, when normally distributed. Skewed data where log transformed, and changes from baseline on the log scale were calculated, and these changes now correspond to a multiplicative change from baseline on the original scale. Subjects were included as a random factor within a linear mixed model. Fixed effects included in the final model were: baseline parameters, treatment, treatment order, and changes in BMI. Further exploratory investigation of equol group was included in the model. Pearson's correlation coefficient was calculated between baseline PYY and changes in PYY. Statistical analysis was performed using SAS 8.4 (SAS Institute Inc., Cary, NC).

## Abbreviations

BMI: body mass index; PYY: peptide YY.

## Competing interests

This study was carried out with financial support from the Commission of the European Communities, ISOHEART QLK1-2001-00221. It does not necessarily reflect its views and in no way anticipates the Commission's future policy in this area. The authors declare that they have no competing interests.

## Authors' contributions

MOW, BO, CK, and MR were responsible for data analysis and writing of the manuscript. S B, CMW, and HJZ were responsible for the study design and were involved in all aspects of the study as well as manuscript review. MR, JH and MF, and KV were involved in the collection of the data. BO, WLH, and DT were responsible for laboratory analysis. All authors contributed to the manuscript.
